# Contained Left Ventricular Free Wall Rupture following Myocardial Infarction

**DOI:** 10.1155/2012/467810

**Published:** 2012-12-03

**Authors:** Arthur Shiyovich, Lior Nesher

**Affiliations:** ^1^Internal Medicine E, Soroka University Medical Center, P.O. Box 151, 84101 Beer Sheva, Israel; ^2^Department of Emergency Medicine, Rekanati School for Community Health Professions, Faculty of Health Sciences, Ben-Gurion University of the Negev, 84105 Beer Sheva, Israel

## Abstract

Rupture of the free wall of the left ventricle occurs in approximately 4% of patients with infarcts and accounts for approximately 20% of the total mortality of patients with myocardial infractions. Relatively few cases are diagnosed before death. Several distinct clinical forms of ventricular free wall rupture have been identified. Sudden rupture with massive hemorrhage into the pericardium is the most common form; in a third of the cases, the course is subacute with slow and sometimes repetitive hemorrhage into the pericardial cavity. Left ventricular pseudoaneurysms generally occur as a consequence of left ventricular free wall rupture covered by a portion of pericardium, in contrast to a true aneurysm, which is formed of myocardial tissue. Here, we report a case of contained left ventricular free wall rupture following myocardial infarction.

## 1. Introduction

Rupture of the free wall of the left ventricle occurs in approximately 4% of patients with myocardial infarction (MI) and accounts for approximately 20% of mortality of these patients [1, 2]. Premortem diagnosis of rupture is made in approximately 15% of in-hospital deaths from acute MI in a coronary care unit [3]. However, one series of autopsies claims that up to 31% of MI fatalities had cardiac rupture. Hence, relatively few cases of left ventricular free wall rupture (LVFWR) are diagnosed before death. Nevertheless, the increased availability of bedside echocardiography has contributed to a progressive rise in the number of cases of LVFWR being diagnosed and reported. Several distinct clinical forms of ventricular free wall rupture have been identified [4]. Sudden rupture with massive hemorrhage into the pericardium is the most common form; in a third of the cases, the course is subacute with slow and sometimes repetitive hemorrhage into the pericardial cavity [5]. Left ventricular pseudoaneurysm is a variant of left ventricular rupture that generally occurs as a consequence of LVFWR covered by a portion of pericardium. Here, we report a case of contained left ventricular free wall rupture following myocardial infarction.

## 2. Patient Description

**Figure 1:**
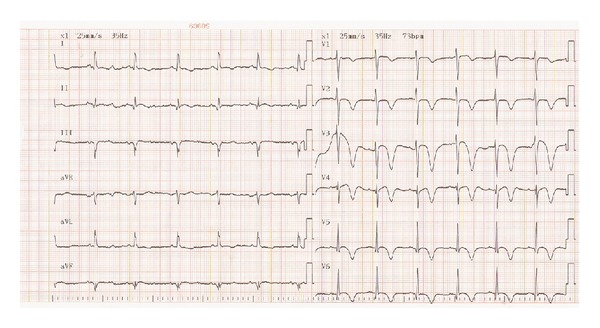
Twelve-lead ECG of the patient: sinus rhythm 73 p/minute, axis of 5 degrees with pathological Q waves in leads V4–V6, ST-segment elevations in leads V1–V3, and T wave inversions in V1–V6 and in leads I and AVL.

**Figure 2:**
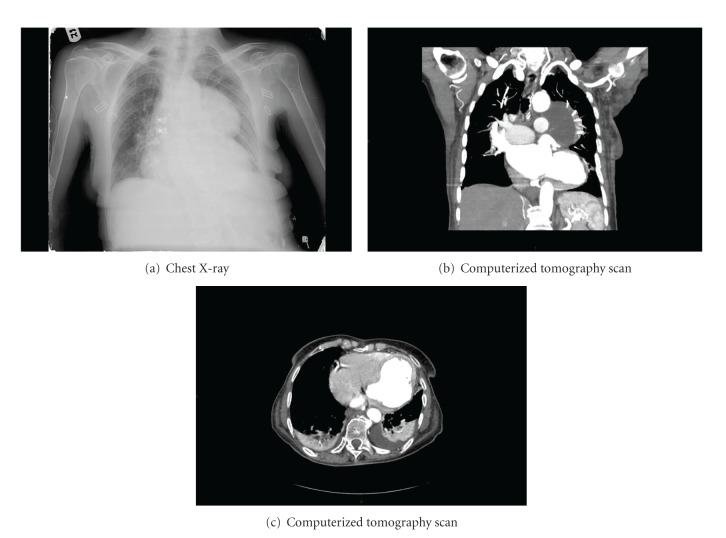
(a) A posterior-anterior X-ray of the chest which reveals a space-occupying lesion with clearly defined borders. The lesion is of single consistency without calcification and merges with the lower border of the heart situated at a wide angle to the shadow of the heart, appearing as a mass in the anterior middle mediastinum. (b) Computerized tomography scan reveals a cystic process in the anterior middle mediastinale region with a double layer of fluid, one of which is consistent with blood. The process merges with the left ventricle. (c) CT Frame 205 of the same exam revealed thinning of the posterior lateral wall of the left ventricle with an aneurysm of approximately 50 millimeters, expanding from the left ventricle.

An 80-year-old retired female resident of a home for the aged was admitted with recent complaints of dyspnea, dizziness, and a falling episode with a possible loss of consciousness. Her personal history revealed mild dementia, Parkinson's disease treated with carbidopa and levodopa, hypertension treated by nifedipine, and dyslipidemia treated by statins. Additional medications included acetylsalicylic acid (100 mg qd), calcium supplements, and brotizolam as a sleep inducer. The patient did not take any other medication or vitamin supplements. At admission to the Emergency Department, the patient's respiratory rate was 16 breaths/min, blood pressure was 96/60 mm Hg, heart rate was regular at 100/min, room-air oxygen saturation was 92% and temperature was 36.7°C. Physical examination revealed no apparent distress, pale 80-year-old Caucasian female, good breath sounds bilaterally, heart sounds were regular and distant, abdomen was nontender, no organomegaly was observed, and pulses were palpated as normal in the radial and femoral points of examination. A 12-lead electrocardiogram obtained in supine posture showed an axis of 5 degrees with pathological Q waves in leads V4–V6, ST-segment elevation in leads V1–V3, and T wave inversion in V1–V6 and in leads I and AVL (Figure 1). Cardiac enzymes were indicative of myocardial injury; troponin T levels were 1.93 ng/mL (normal 0–0.014); creatine kinase was 9684 U/L (normal 20–180 U/L); lactate dehydrogenase was 1443 U/L (normal 230–480); myoglobin was 1865 ng/mL (normal 19–51 ng/mL). Creatinine levels were 0.71 mg/dL (normal 0.51–0.95 mg/dL); urea was 57 mg/dL (normal 17–43 mg/dL). Liver function tests were mildly elevated. Aspartate aminotransferase was 217 U/L (normal 0–31 U/L); alanine transaminase was 84 (normal 0–34). Sodium was 129 mEq/L (normal 125–135), otherwise the complete blood count and electrolytes were all within normal limits. Chest X-ray image (Figure 2(a)) revealed a space-occupying lesion with clearly defined borders. The lesion was of single consistency without calcification. The lesion merged with the lower border of the heart situated at a wide angle to the shadow of the heart, appearing as a mass in the anterior middle mediastinum. Cranial computerized tomography (CT) scan, performed to rule out intracranial hemorrhage related to the falling episode, was interpreted as normal. The patient was admitted to a monitored bed. A chest CT scan with contrast material was performed. Frame 143 of the scan revealed a cystic process in the anterior middle mediastinal region with a double layer of fluid lucency, one of which is consistent with blood. The process merged with the left ventricle. CT Frame 205 of the same exam revealed thinning of the posterior lateral wall of the left ventricle with an aneurysm of approximately 50 millimeters, expanding from the left ventricle (Figure 2(b)). A transthoracic echocardiogram was performed that demonstrated severe left ventricular dysfunction, severe mitral regurgitation, moderate-to-severe pulmonary hypertension, and a huge left ventricular lateral wall aneurysm.

At this point, it was evident that the patient had a contained left ventricular free wall rupture following myocardial infarction. Following a detailed explantation of the treatment options to the patient and her family, a noninvasive/nonsurgical treatment modality was chosen. Combined pharmacological therapy including acetylsalicylic acid (aspirin 100 mg), ACE inhibitor (ramipril 5 mg), diuretic (lasix 40 mg), and beta blocker (carvedilol 6.25 mg × 2) improved the patient's hemodynamic status and reduced the symptoms of heart failure (mostly dyspnea), additional treatment included a statin (simvastatin 20 mg), and a benzodiazepine for sleeping (britazolam 0.25 mg). After six days of hospitalization, the patient was discharged for ambulatory followup. Approximately two months following discharge, the patient was admitted with exacerbation of the heart failure complaints. Echocardiography demonstrated no change in the aneurysm or the ventricular function. Following an increase in the dose of the diuretics and stabilization of the symptoms, home oxygen was arranged and the patient was discharged.

## 3. Comment

Ventricular free wall rupture occurs up to ten times more frequently than septal or papillary muscle rupture [6]. It progresses from the endocardium to the pericardium and occurs through an area of necrosis. Risk factors for rupture include hypertension, older age, female sex, first infarction, anterior infarction, and large transmural infarction of at least 20% [7]. In the case considered here most of the risk factors were present. 

Most ventricular ruptures occur within the first week after MI, approximately 50% within the initial four days; however, some cases have been reported as late as one month or even later. Acute LVFWR may present by chest pain, or by the classic features of cardiac tamponade, namely, shock with hypotension, pulsus paradoxus, elevated venous pressure, quiet heart sounds, sinus bradycardia, or frank electromechanical dissociation. Death usually ensues in a matter of minutes to hours. Cardiopulmonary resuscitation maneuvers are uniformly unsuccessful in these cases. 

In the subacute form, the presentation may evolve over hours, days, or even longer. This form usually presents mainly with pericardial effusion signs and symptoms and may present with dysrhythmias, syncope, prolonged or recurrent chest pain (sometimes of the pericardial type), and heart failure [8]. In this form, the rupture is often sealed by the epicardium or alternatively by a haematoma in the epicardial surface of the heart, forming a contained myocardial rupture. Pathologically, this situation stands somewhere between free rupture into the pericardial cavity and formation of pseudoaneurysm [7].

Opinions differ as to the most common site of the left ventricular rupture. It was suggested that a lateral wall infarction is more likely to rupture than an anterior or inferior infarction. However, anterior MIs are more frequent than lateral MIs and thus the anterior wall is the most common site. Another possible explanation for the greater prevalence of posterior subacute ruptures is that ruptures of the anterior wall cannot be tolerated, as they are rarely compressed by an adherent pericardium. The inflammatory reaction of the posterior pericardium might result in pericardial adhesions and formation of a posterior left ventricular pseudoaneurysm.

Electrocardiographic findings in LV rupture patients may be related to its type and severity. Electromechanical dissociation and bradycardia are features of the acute variety, while new ST elevation (“saddle shaped”) in the affected leads or persistent noninversion of T waves may suggest the less noisy “stuttering” type of rupture.

Echocardiography (transthoracic or transesophageal) is usually the preferred imaging test when LVFWR is suspected and is considered highly accurate. Color flow imaging and pulsed Doppler may be useful in the assessment of flow characteristics at the presumed rupture site, and an intravenous echocardiographic contrast agent may be useful in identifying intrapericardial hemorrhage caused by myocardial rupture or the development of ventricular pseudoaneurysm. The most common finding is pericardial effusion and its absence excludes the diagnosis of ventricular rupture.

Recent evidence indicates that cardiac magnetic resonance imaging (MRI), especially with contrast enhancement, is emerging as a valuable diagnostic tool providing visualization of the entire heart and clear differentiation of structures such as the pericardium, myocardium, thrombus, and epicardial fat [9, 10]. Clearly MRI is not the investigation of choice in acute patients with hemodynamic instability; however, in subacute cases, timely contrast-enhanced MRI may help to delineate the anatomical location of FWR and aneurismal change, thereby enabling planned surgical intervention. Additionally, it may identify areas of ischemic myocardium even in the absence of definite ECG findings and myocardium at risk of impending rupture. 

Multidetector computed tomography has also been shown to be effective in detecting LVFWR and may assist in the diagnosis [11, 12], especially when MRI is not available or when time is imperative.

Early diagnosis is imperative in these cases as many ventricular ruptures may be surgically corrected with a good long-term outcome. Although it is a generally acceptable that surgery provides the only definitive treatment of subacute LVFWR [13], cases of patients with long-term survival following medical management have been reported. Medical management usually includes avoidance of obstipation, prolonged bed rest, strict blood pressure control (preferably with beta blockers), and pericardiocentesis as needed. The goals of surgery should be to stop bleeding, to relieve cardiac tamponade, and to prevent a second rupture. Multiple surgical techniques have been described but all involve extensive debridement into normal muscle, thrombectomy of the ventricle, and closure with or without a patch, with preservation of left ventricular geometry. Coronary angiography is also warranted in cases of ventricular rupture, in order to determine whether coronary artery bypass grafting is needed in addition to the repair of the rupture [13].

The overall hospital mortality in patients with and without surgery was reported to be approximately 60% [14]. Surgery-related mortality is up to 33% but in those who survive the complicated operation, the long-term outcome is good. 

In conclusion, it remains clear that despite the significant risk, surgery is the cornerstone treatment of subacute LVFWR. Nevertheless, nonsurgical management may be considered for carefully selected patients, especially for those at high surgical risk. We believe it is imperative for physicians in various specialties to be well acquainted with this entity to be able to consider it in the differential diagnosis while there is still time to save these patients' lives.
